# Effects of tonic inhibition on a cortical neuronal population: implications for general anesthesia under propofol

**DOI:** 10.1186/1471-2202-14-S1-P178

**Published:** 2013-07-08

**Authors:** Laure Buhry, Axel Hutt

**Affiliations:** 1 NEUROSYS team, INRIA Nancy - Grand Est, Villers-lès-Nancy, 54600, France

## 

Anaesthetic propofol, commonly used for general anaesthesia, is known to bind to GABAA receptors [1]. It affects both synaptic and extra-synaptic receptors [2]; inducing a phasic inhibition in the first case and tonic inhibition in the latter case [3]. The effects of phasic inhibition on neuronal populations have been extensively studied, including in the case of propofol-anesthesia; however the influence of tonic inhibition on neuronal population remains an open question and changes in EEG recordings of anesthetized animals cannot be completely explained by phasic inhibition. This is the case for phenomena that appear in occipital recordings and also correlated with the loss of consciousness. Contrarily to frontal recordings that show increased alpha-rhythms (8-12 Hz) that can be modeled with phasic inhibition [4], occipital EEGs exhibit a) a decrease of alpha rhythms concurrently to b) an increase of slow delta rhythms (0-4 Hz). Our question is then: Can tonic inhibition elicit a) and b) in a cortical population of neurons?

To answer this question, we built a model of cortical population made of 750 excitatory and 250 inhibitory neurons whose dynamics resemble those found in biological neurons. Excitatory cells are modeled by a leaky-integrate-and-fire model of pyramidal neurons [5] and inhibitory cells by a Morris-Lecar model [6]. The cells are connected by exponential synapses with time constants of 5 ms for excitatory synapses and 20 ms for inhibitory ones. Tonic inhibition is represented by a maximal conductance and an equilibrium potential close to the resting state of the cell.

We show with our minimal model of a cortical assembly that the peak in the power spectrum of the mean membrane voltage of excitatory cells, whose spiking frequency originally is about 11 Hz without tonic inhibition, shifts towards slower frequencies with increasing tonic inhibition. This result is similar to occipital EEG recordings under propofol anesthesia. However, the amplitude of the power spectra diminishes drastically, more so than in biological recordings, in the presence of tonic inhibition compared to without tonic inhibition. This may indicate that tonic inhibition is not the only occurrence responsible for the increase in amount of delta oscillations, but that thalamic inputs may help maintaining slow cortical occipital activity under general anesthesia. We are thus planning for future works to include interactions in our model between cortical and thalamic populations.
